# Detailed analysis of recovery process of cranial nerve palsy after IMRT-based comprehensive treatment in nasopharyngeal carcinoma

**DOI:** 10.1186/s13014-021-01846-x

**Published:** 2021-06-27

**Authors:** Jian Zang, Yan Li, Shanquan Luo, Jianhua Wang, Bingxin Hou, Min Yao, Lina Zhao, Mei Shi

**Affiliations:** 1 Department of Radiation Oncology, Xijing Hospital, Fourth Military Medical University, No. 127, Chang Le West Road, Xi’an, 710032 China; 2 Department of Radiation Oncology, University Hospitals Cleveland Medical Center, Cleveland, USA

**Keywords:** Cranial nerve palsy, IMRT, Nasopharyngeal carcinoma, Comprehensive treatment, Recovery

## Abstract

**Background:**

Cranial nerve (CN) palsy due to cancer involvement has been considered as an unfavorable prognostic factor for patients with nasopharyngeal carcinoma (NPC). We assessed the role of IMRT based treatment on the recovery of CN palsy and investigated the prognostic value of complete recovery of CN palsy.

**Methods:**

A total of 115 NPC patients with cancer-related CN palsy were included in the study. We referred CTCAE version 5.0 to evaluate the grade of CN palsy.

**Results:**

All patients with grade 1 CN palsy recovered completely during the 2 years of follow-up after definite treatment. Most grade 2 palsy could change gradually to grade 1 palsy or complete recovery during 2 years of follow-up. Patients with more than 2 symptoms of CN palsy had poor 3-year disease-free survival (DFS) than these with 1 or 2 symptoms (60.3% vs. 84.9%, HR 0.25, 95% CI 0.07–0.89, *P* = 0.001). There were no significant differences for PFS, OS, DMFS and LRFS between patients with complete recovery and non-complete recovery from CN palsy after receiving IMRT based comprehensive treatment.

**Conclusions:**

IMRT based comprehensive treatment could effectively promote the recovery of tumor-related CN palsy for NPC patient. More than 2 symptoms of CN palsy was a poor prognostic factor for DFS of NPC patients. The prognostic role of complete recovery of CN palsy was not identified in our study.

**Supplementary Information:**

The online version contains supplementary material available at 10.1186/s13014-021-01846-x.

## Introduction

Nasopharyngeal carcinoma (NPC) is a cancer arising from the nasopharyngeal mucosal lining, from where the tumor can invade adjacent anatomical organs. Hence, depending on the involvement of anatomical structures, the clinical symptoms varies considerably from person to person, including epistaxis, nasal obstruction, hearing impairments, headache and cranial nerve (CN) palsy. The incidence of CN palsy is approximate 8 to 12.4% in NPC [1]. CN palsy is classified as T4 stage according to the American Joint Committee on Cancer/Union for International Cancer Control (AJCC/UICC). It has been considered as an unfavorable prognostic factor for NPC patients [2, 3]. Several studies reported patients with complete recovery from CN palsy had better overall survival (OS) than these without complete recovery after receiving conventional radiation technique or 3-dimensional conformal radiation therapy (3D-CRT) based comprehensive treatment. Moreover, multiple CN palsy and long recovery duration were associated with poor OS [4, 5]. Therefore, the recovery of CN palsy seems to predict a better survival outcome before intensity modulated radiation therapy (IMRT) era. However, the detail of recovery process of CN palsy has not been investigated by these studies.

Undoubtedly, IMRT is a “Game-Changing” radiation technique for NPC due to the improved dosimetric properties. Locoregional control and survival have been improved by IMRT, and toxicity has been reduced [6, 7]. Compared with conventional radiotherapy and 3D-CRT, IMRT could improve the local control from 84.7 to 90.5% for NPC [7]. Induction chemotherapy followed by IMRT based concomitant chemoradiotherapy (IC + CCRT) and concomitant chemoradiotherapy (CCRT) are alternative treatment options for patients with CN palsy according to National Comprehensive Cancer Network (NCCN) guidelines and guidelines of Chinese Society of Clinical Oncology (CSCO). These treatment modalities result in more than 90% local control rate for patients with locoregionally advanced NPC [8, 9]. However, it is still unknown whether the superior local control rate could lead to a high relief rate of CN palsy. In this study, we investigate the recovery process of different symptoms of CN palsy, and explore the influence of treatment-related factors on the recovery of CN palsy and the prognostic value of CN palsy recovery in the era of IMRT. To our knowledge, this is the first study focus on the detailed recovery process of CN palsy in NPC patients receiving IMRT-based treatment modalities.

## Methods and materials

### Patients

We reviewed the NPC database from cancer-specific database platform in XiJing Hospital between January 1, 2012 and December 31, 2016 and 514 patients with histologically proved nasopharyngeal squamous cell carcinoma treated with radiotherapy were identified. Of these patients, 121 (22.4%) had CN palsy symptoms. Six patients were excluded: 2 received radiotherapy alone, 3 did not complete the treatment course, and 1 received palliative treatment. The remaining 115 NPC patients with CN palsy were included in the study. This retrospective review was approved by our institutional review board and was in accord with the Helsinki Declaration of 1975 as revised in 1983.

### Diagnosis and evaluation

The diagnosis of CN palsy depended on clinical symptoms physical examination, and magnetic resonance imaging (MRI) with or without thin slice computerized tomography imaging (CT) of skull base. CN invasion was detected on MRI that met one of the following criteria: (1) enhancing soft-tissue tumor along the course of the ipsilateral-related nerve, replacing the normal structures of the CN on gadolinium-enhanced T1-weighted images; or (2) perineural spread, defined as an enlargement or abnormal enhancement of the nerve, obliteration of the neural fat pads adjacent to the neurovascular foramina, and neuroforaminal enlargement [10, 11]. Physical examinations of CN palsy were conducted by two radiation oncologists every time from pretreatment to the whole follow-up period. Two radiologists reviewed all the imaging records and disagreements were resolved by consents. CN palsy was defined as cranial nerve invasion detected by imaging combined with nerve palsy symptoms resulted by tumor invasion. Because of lack of criteria for CN palsy grade, we referred Common Terminology Criteria for Adverse Events (CTCAE) version 5.0 to evaluate the grade of CN palsy. Grade 1 palsy was defined as asymptomatic, clinical or diagnostic observation only and intervention not indicated. Grade 2 palsy was defined as moderate symptoms with limiting instrumental activities of daily living. Graded 3 palsy was defined as sever symptoms with limiting selfcare.

### Treatment

All patients were treated with definitive radiotherapy. The detail of radiotherapy process was described in our previous studies [12, 13]. The selections of radiation dose for patients depend on tumor volume, response to induction chemotherapy, age and (Eastern Cooperative Oncology Group, ECOG) score. Patients with higher primary tumor volume (> 55.5 ml) and less than 50% tumor regression after induction chemotherapy would receive higher radiation dose. In this study, 34 of 115 patients (29.6%) received fixed-gantry IMRT technique, 81 (70.4%) patients received volumetric modulated arc therapy (VMAT) technique.

Of these patients, 63 (54.8%) were treated with IC followed by CCRT, and 52 patients (45.2%) received CCRT alone. IC was administrated to patients with high risk metastatic lymph nodes, such as N2 to N3, extracapsular nodal spread, central nodal necrosis, ≥ 3 cm in greatest dimension, and nodal grouping metastasis. Patients with N0 or low risk N1 received CCRT alone. The induction chemotherapy included TP regimen (docetaxel 75 mg/m^2^, cisplatin 75 mg/m^2^), PF regimen (cisplatin 80 mg/m^2^, 5-FU 800–1000 mg/m^2^ days 1 to 5), GP regimen (gemcitabine 1000 mg/m^2^, cisplatin 75 mg/m^2^), and TPF regimen (docetaxel 75 mg/m^2^, cisplatin 75 mg/m^2^, 5-FU 750 mg/m^2^ days 1 to 5) given every 3 weeks for 2–3 cycles. Concurrent chemotherapy consisted of cisplatin (100 mg/m^2^ every 3 weeks or 40 mg/m^2^ weekly).

### Definition and statistical analysis

Complete recovery was defined as complete disappearing of CN palsy symptoms. The follow up time was calculated from the date of treatment completion to either the date of death or the last review date. Patients were reexamined at least every 3 months in the first 2 years, then every 6 months for up to 5 years or until death. The grade of CN palsy symptoms was recorded in detail by two radiation oncologists. The survival endpoints included overall survival (OS), progression-free survival (PFS), distant metastasis-free survival (DMFS) and locoregional recurrence-free survival (LRFS). OS was measured from the date of treatment completion to death; PFS was measured from the date of treatment completion to the date of disease progression or death from any causes; DMFS was defined as the time from end of treatment to first detection of distant metastasis; LRFS was defined as the time from end of treatment to first detection of local or lymph node region relapse.

The variation of measurement data in groups were compared by means of Student’s t test, and data was presented as means ± standard error of the mean (SEM) values. Differences in proportions between groups were evaluated by χ^2^ test. Numerical variable was transformed to categorical variable using median as cut-off if it was not Gaussian distribution, such as tumor volume. The Kaplan–Meier method was used to estimate the endpoints, survival curves were compared using the log-rank test. Cox proportional hazard model was used to identify potentially independent prognostic factors, and the proportional hazards assumption was tested with Schoenfeld residuals. The hazard ratio (HR) and its 95% confidence interval (95% CI) were used to indicate the prognostic value of risk factors. A two-sided *P* value of less than 0.05 was considered significant.

## Results

### Patient characteristics and incidence of CN palsy

Patient characteristics are summarized in Table 1. The male/female ratio was 3.8:1. Median age was 49 years (range 18–83 years); 83.5% patient ages were less than 60 years. Most patients (77.4%) had regionally metastatic lymph nodes at the time of diagnosis of NPC. All patients had non-keratinizing squamous cell carcinoma. Sixty-three patients (54.8%) received induction chemotherapy. The median radiation dose was 72.62 Gy.

**Table 1:** Clinical characteristics of 115 patients with CN palsy

Characteristics	N (%)
Sex	
Male	91 (79.1)
Female	24 (20.9)
Age	Median: 49 years (range 18–83 years)
N stage	
N0	26 (22.6)
N1	49 (42.6)
N2	35 (30.4)
N3	5 (4.4)
Histological type	
Non­keratinising differentiated	37 (32.2)
Non­keratinising undifferentiated	78 (67.8)
Treatment regimen	
IC + CCRT	63 (54.8)
CCRT	52 (45.2)
Radiation dose of primary tumor	Median: 72.62 Gy (range 67.2–78.8 Gy)
≤ 72.62 Gy	57 (49.6)
> 72.62 Gy	58 (50.4)
Tumor volume of primary tumor	Median: 55.5 ml (range 4.11–232 ml)
≤ 55.5 ml	58 (50.4)
> 55.5 ml	57 (49.6)
CN palsy symptom	
Trigeminal nerve disorder	104 (90.4)
Abducens nerve disorder	42 (36.5)
Oculomotor nerve disorder	26 (22.6)
Hypoglossal never disorder	7 (6.1)
Facial nerve disorder	4 (3.5)

IC + CCRT, induction chemotherapy followed by concurrent chemoradiotherapy; CCRT, concurrent chemoradiotherapy; CN, cranial nerve

Trigeminal nerve (TN, CN V) disorder was the most common CN palsy symptom (90.4%), followed by abducens nerve (AN, CV VI) disorder (36.5%), oculomotor nerve (ON, CN III) disorder (22.6%), hypoglossal nerve (HN, CN XII) disorder (6.1%) and facial nerve (FN, CN VII) disorder (3.5%). Of all patients, 68 patients (59.1%) had one symptom of CN palsy, 30 patients (26.1%) had two symptoms of CN palsy and 17 patients (14.8%) had more than three symptoms of CN palsy. Because the incidences of HN disorder and FN disorder were very low, we just focused on the recovery of TN disorder, AN disorder and ON disorder in this study.

### Correlation between duration of CN palsy and symptom grade

**Fig. 1:**
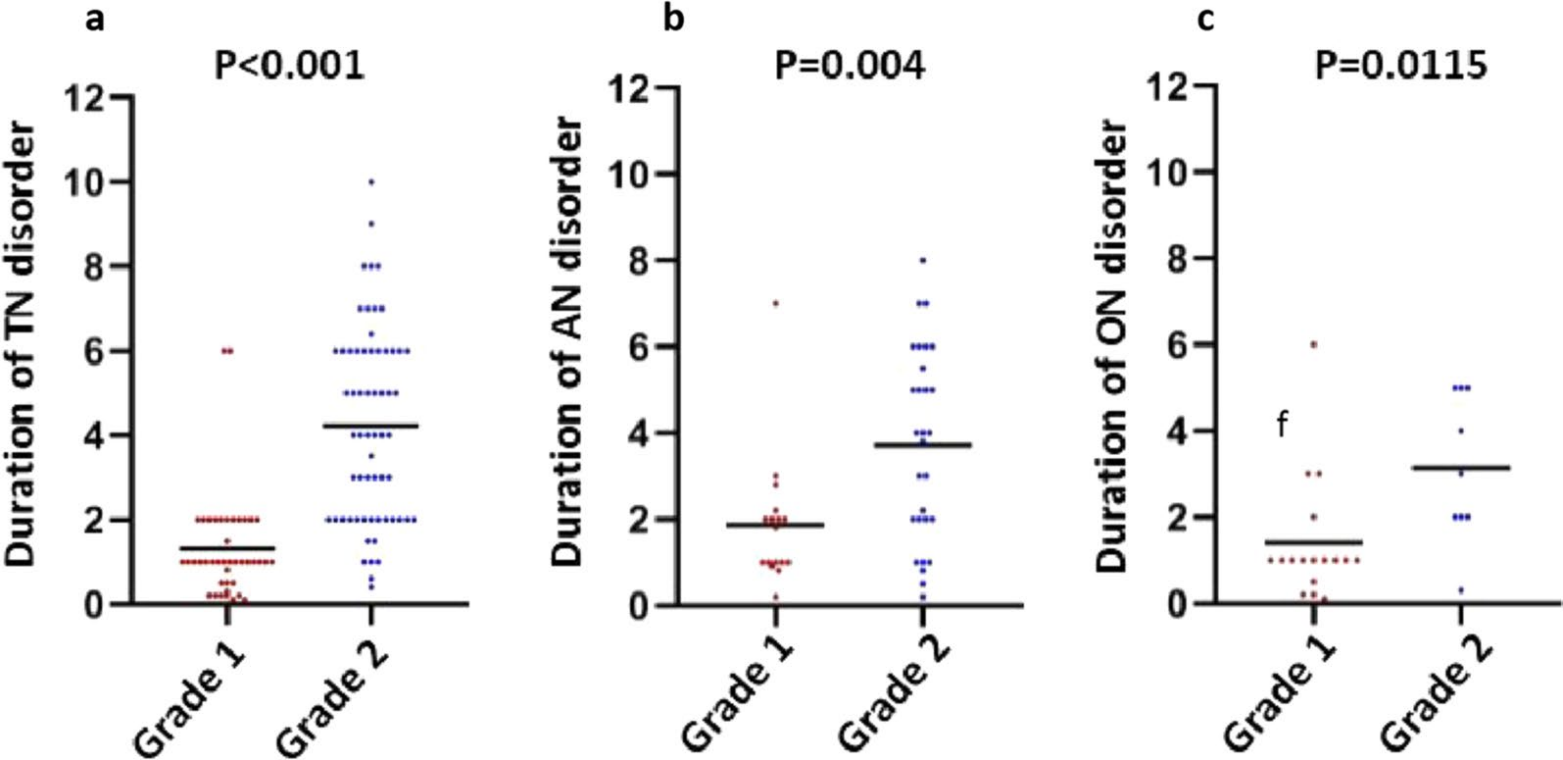
Correlation between grade and symptom duration of cranial palsy. TN, trigeminal nerve (**a**); AN, abducens nerve (**b**); ON, oculomotor nerve (**c**)

According to the grade definition of CN palsy, no patients had grade 3 symptom in this cohort. Patients with grade 2 CN palsy had significantly longer duration of symptom before clinical diagnosis. The mean duration time were 4.22 ± 0.29 months versus 1.32 ± 0.19 months between patients with grade 2 and grade 1 TN disorder, 3.72 ± 0.41 months versus 1.86 ± 0.37 months between grade 2 and grade 1 AN disorder, 3.14 ± 0.57 months versus 1.41 ± 0.35 months between grade 2 and grade 1 ON disorder, with significantly statistical differences (Fig. 1).

### Evaluation of recovery process of CN palsy

**Fig. 2:**
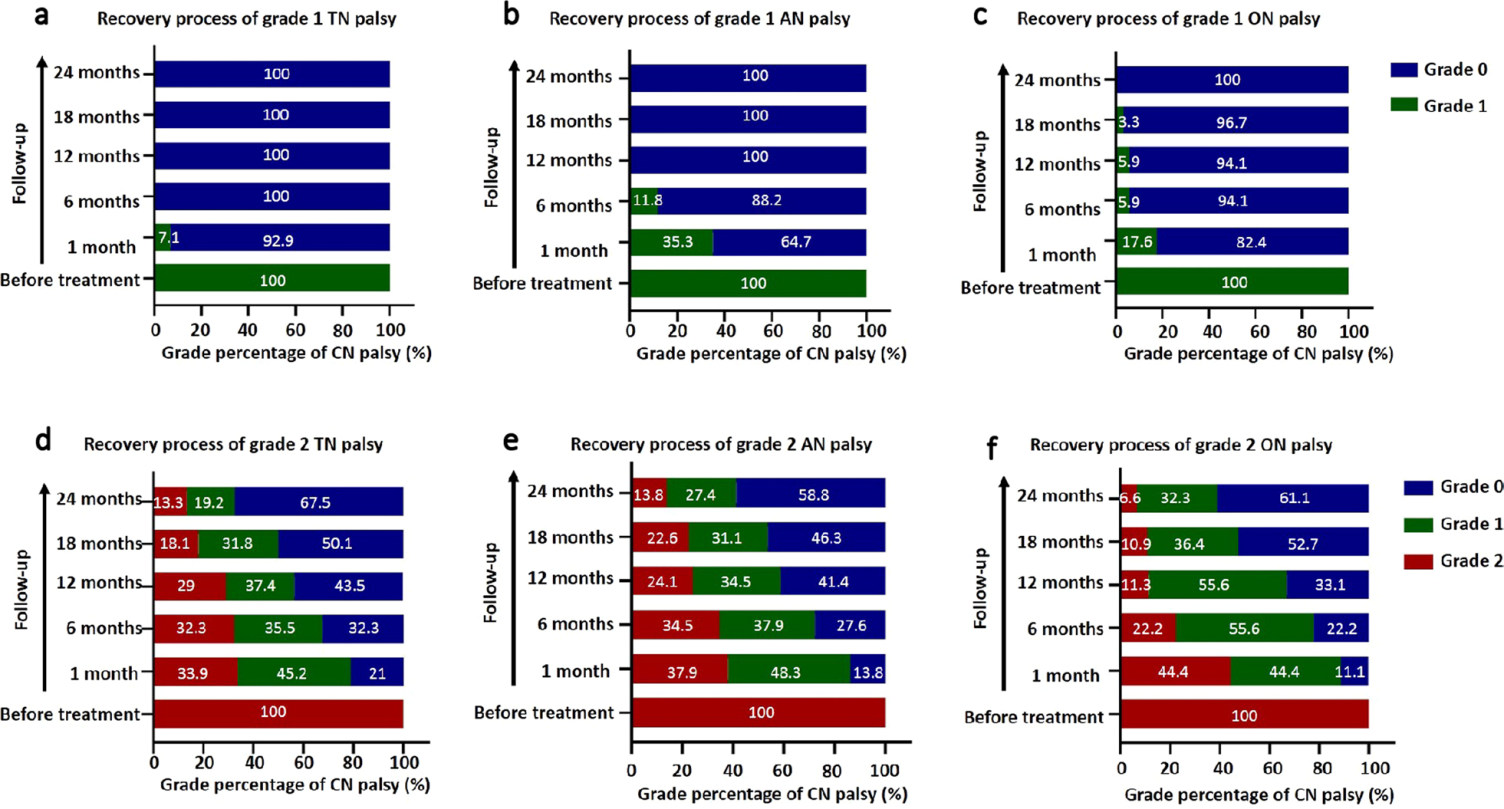
Grade changing of cranial palsy after completion of treatment. TN, trigeminal nerve (**a**, **d**); AN, abducens nerve (**b**, **e**); ON, oculomotor nerve (**c**, **f**)

In general, the palsy severity of TN, AN and ON were reducing gradually after completion of treatment. During 2 years of follow-up, the proportion of patient with any grade of TN, AN, and ON palsy decreased from 90.4 to 20%, 36.5 to 8.3%, 22.6 to 5%, respectively. The rate of complete recovery of TN, AN, and ON palsy were 77.9%, 77.2% and 77.8% during the 2-years follow-up (Additional file 1: Fig. S1). The grade of CN palsy before treatment had an impact on the recovery of CN symptom. All patients with grade 1 CN palsy recovered completely during the 2 years of follow-up after definite treatment (Fig. 2a–c). Most grade 2 TN, AN and ON palsy could change gradually to grade 1 palsy or complete recovery during 2 years of follow-up (Fig. 2d–f). The complete recovery rate of grade 2 TN, AN, and ON palsy were 67.5%, 58.8%, and 61.1%, respectively. However, there was still a small percentage of patients who had persistent grade 2 TN (13.3%), AN (13.8%), and ON (6.6%) palsy (Fig. 2d–f).

### Influence of treatment-related factors on complete recovery of CN palsy

**Fig. 3:**
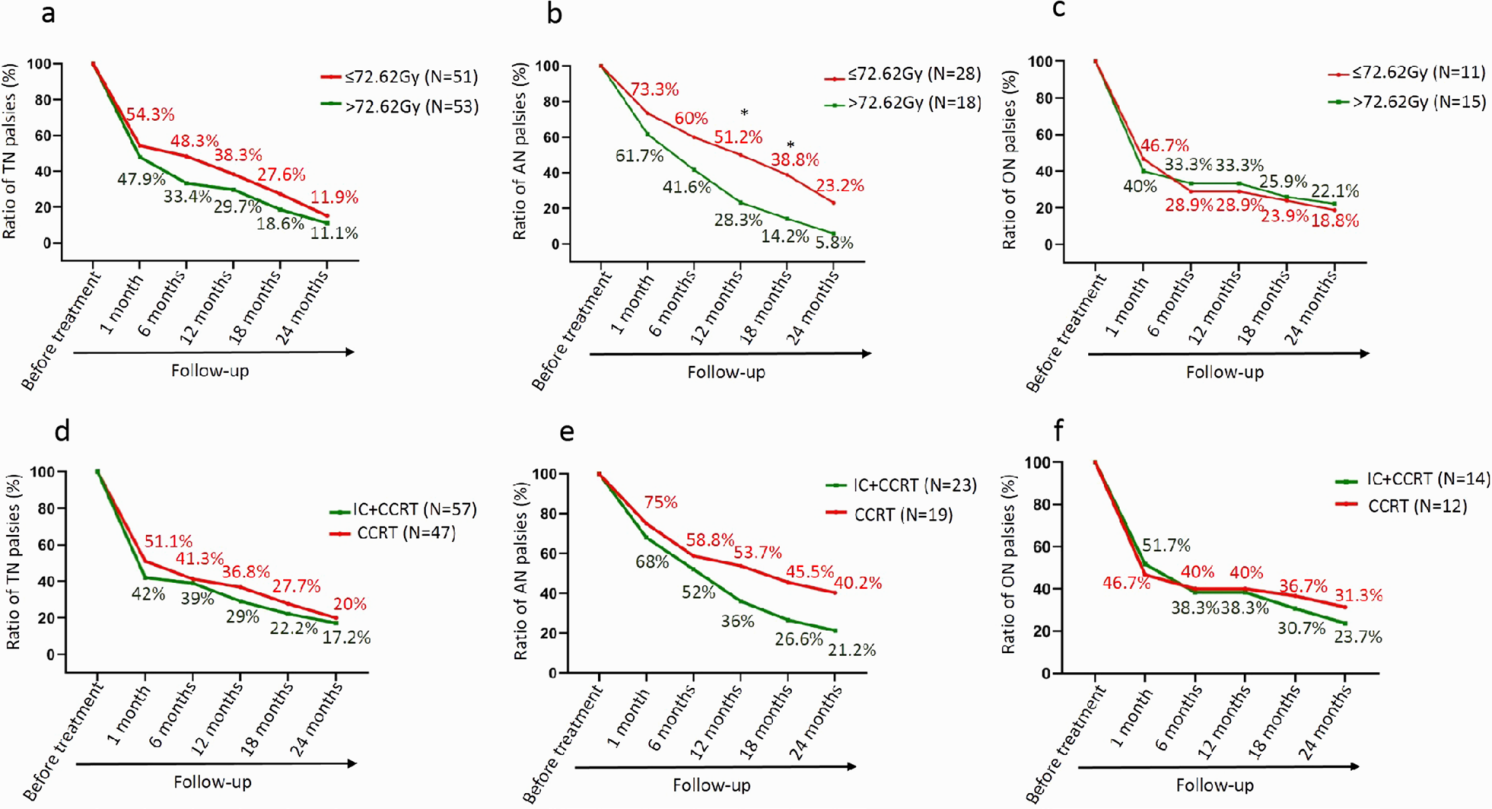
Impact of treatment-related factors on recovery of cranial nerve palsy. **a**–**c** Impact of radiation dose on recovery of cranial nerve palsy. Median radiation dose was used as dichotomy; **d**–**f** impact of treatment modalities on recovery of cranial nerve palsy. TN, trigeminal nerve; AN, abducens nerve; ON, oculomotor nerve

Treatment-related factors associated with complete recovery of CN palsy were explored including radiation doses and different treatment modalities. Using median radiation dose (72.62 Gy) as dichotomy, patients were divided into two groups: low radiation dose groups (≤ 72.62 Gy) and high radiation does group (> 72.62 Gy). There were no significant differences in number of CN palsy symptom between the groups of radiation dose (data was not shown). For grade 2 of TN palsy, there were more patients received radiotherapy with low radiation dose than those received high radiation dose (58.1% vs. 41.9%, *P* = 0.049). For grade 2 of AN palsy, there were more patients received radiotherapy with low radiation dose than those received high radiation dose (65.5% vs. 34.5%, *P* = 0.047). For grade 2 of ON palsy, the proportions of patients received low radiation dose and high radiation dose were not different (44.4% vs. 55.6%, *P* = 0.749). After comparison of recovery rate between the groups of radiation dose, there were no differences of complete recovery rates of TN palsy and ON palsy between two radiation dose groups (Fig. 3a, c). However, for AN palsy, patients received higher radiation dose (> 72.62 Gy) had higher complete recovery rate than those received lower radiation dose (≤ 72.62 Gy), with significantly statistical differences between two groups in 12 months (28.3% vs. 51.2%) and 18 months (14.2% vs. 38.3%) after treatment (Fig. 3b).

There were no significant differences in complete recovery rates of TN palsy and ON palsy between patients treated with IC + CCRT versus CCRT. Compared with CCRT, IC + CCRT had a trend to improve the recovery rate of AN palsy, but not reached significant difference (Fig. 3d–f).

### Prognostic value of CN palsy

The median follow-up was 48 months (range 7–109 months). For the whole cohort, the 3-year and 4-year DFS, OS, DMFS and LRFS were 80.9%, 88%, 89.4%, 97.1% and 78.6%, 84%, 88.2%, 94.4%, respectively.

**Fig. 4:**
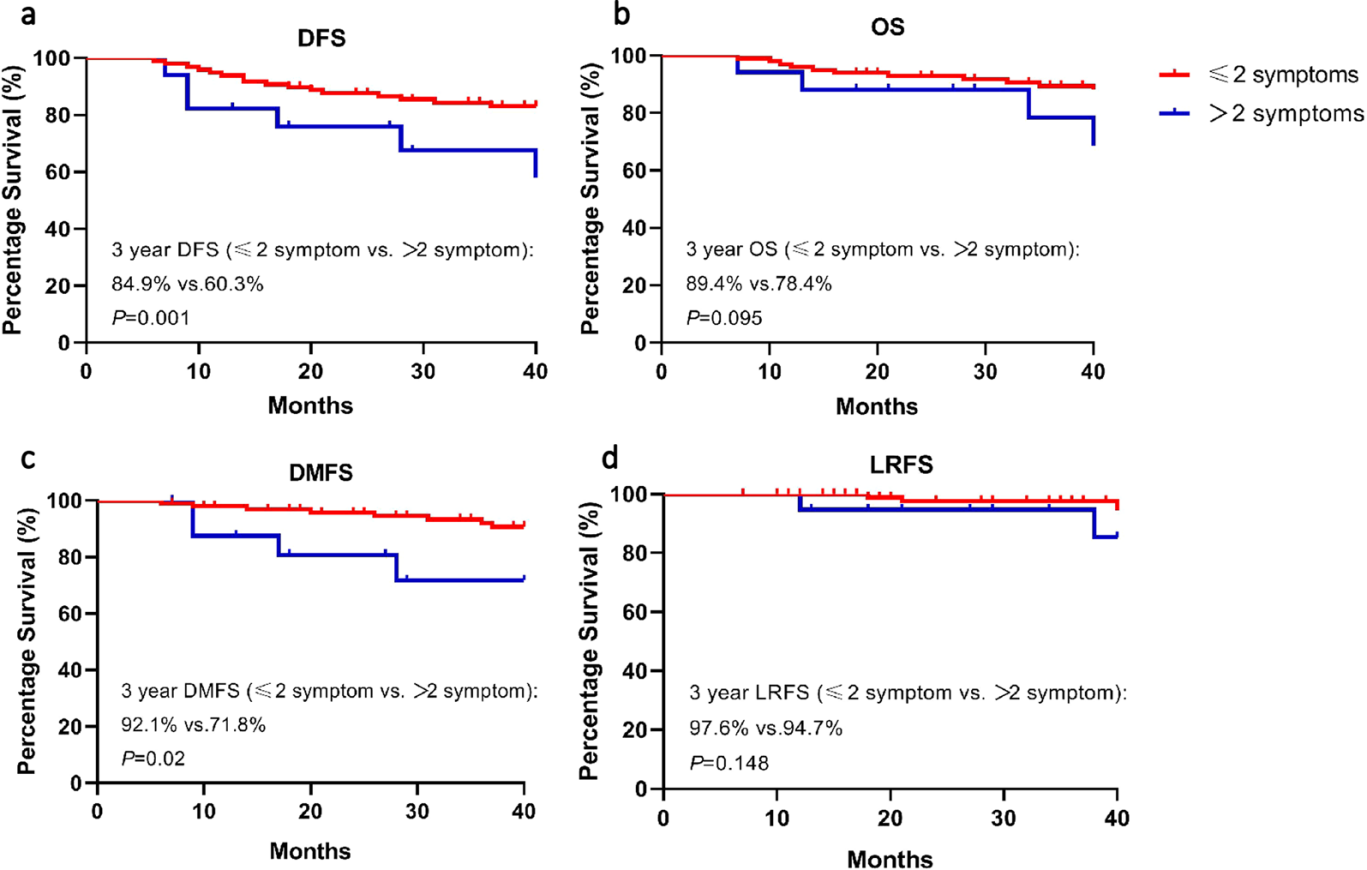
Survival analysis between patients with 1 or 2 symptoms of cranial palsy and patients with more than 2 symptoms of cranial palsy. DFS (**a**), disease-free survival; OS (**b**), overall survival; DMFS (**c**), distant metastasis-free survival; LRFS, locoregional recurrence-free survival (**d**)

In order to clarify the prognostic value of CN palsy, patients were divided into two groups according to the number of CN palsy symptoms they experienced before treatment. Group 1 included patient experienced 1 or 2 symptoms of CN palsy, and patients in group 2 had more than 2 symptoms of CN palsy. The 3-year DFS were 84.9% in group 1 compared with 60.3% in group 2 (HR 0.25, 95% CI 0.07–0.89, *P* = 0.001). Patients in group 1 had a trend in achieving a better 3-year OS compared with those in group 2, but without statistically significant differences (89.4% vs. 78.4%; HR: 0.21, 95% CI 0.06–1.26, *P* = 0.095). Patients in group 1 had significantly better 3-year DMFS than those in group 2 (92.1% vs. 71.8%; HR 0.12, 95% CI 0.02–0.73, *P* = 0.02) (Fig. 4a–c). Nevertheless, there were similar 3-year LRFS between two groups (97.6% vs. 94.7%, HR 0.21, 95% CI 0.02–2.09, *P* = 0.148) (Fig. 4d).

The prognostic value of pretreatment duration of CN palsy was analyzed in different types of CN palsy. A significant difference was observed for DFS (*P* = 0.046) in patients with pretreatment duration within 2 months of TN palsy compared with those with longer than 2 months (Additional file 2: Fig. S2a), but no significant differences were observed for OS (*P* = 0.252), DMFS (*P* = 0.09) and LRFS (*P* = 0.052) between two groups (Additional file 2: Fig. S2b–d). Prognostic value of pretreatment duration of AN and ON palsy were not detected for every survival endpoint (Additional file 3: Fig. S3, Additional file 4: Fig. S4). There were no significant differences for PFS, OS, DMFS and LRFS between patients with complete recovery and non-complete recovery from CN palsy after receiving IMRT based comprehensive treatment (Additional file 5: Fig. S5).

Multivariate analysis was performed to evaluate the potential prognostic factors for every endpoint. Factors included sex, age (≤ 50 years vs. > 50 years), N stage (N0-1 vs. N2-3), histological type, treatment modalities, radiation dose (≤ 72.62 Gy vs. > 72.62 Gy), tumor volume (≤ 55.5 ml vs. > 55.5 ml), number of CN palsy symptom (≤ 2 symptoms vs. > 2 symptoms), initial CN palsy (yes vs. no) and 1-year complete recovery rate of CN palsy (yes vs. no). N stage was a positive prognostic factor for DFS (HR 3.382, 95% CI 1.13–10.13, *P* = 0.029) and DMFS (HR 6.654, 95% CI 1.08–41.06, *P* = 0.041). N stage and age were independent prognostic factors for OS (N stage, HR 4.476, 95% CI 1.02–19.73, *P* = 0.048; age, HR 3.76, 95% CI 1.11–12.79, *P* = 0.034). For LRFS, multivariate analysis failed to detect any positive prognostic factor (Table 2).

**Table 2:** Multivariate analysis of prognostic factors

Factors	HR	95% CI	*P* value
Disease-free survival			
Sex	1.55	0.39–6.07	0.526
Age	2.04	0.76–5.48	0.157
N stage	3.38	1.12–10.28	0.029*
Histological type	2.89	0.88–9.53	0.08
Treatment	0.96	0.38–2.46	0.934
Radiation dose	1.48	0.55–3.94	0.435
Tumor volume	1.69	0.62–4.59	0.307
Number of CN palsy symptom	1.92	0.69–5.28	0.205
Initial TN symptom	0.86	0.21–3.59	0.85
Initial AN symptom	0.95	0.16–5.65	0.963
Initial ON symptom	0.41	0.02–6.98	0.54
1-year recovery rate of TN palsy	3.05	0.68–5.28	0.205
1-year recovery rate of AN palsy	0.63	0.07–5.1	0.665
1-year recovery rate of ON palsy	3.91	0.24–63.48	0.34
Overall survival			
Sex	0.87	0.16–4.55	0.86
Age	3.76	1.11–12.79	0.034*
N stage	4.476	1.02–19.73	0.048*
Histological type	2.98	0.78–11.44	0.111
Treatment	2.57	0.82–8.04	0.106
Radiation dose	0.943	0.27–3.29	0.926
Tumor volume	3.501	0.9–13.61	0.071
Number of CN palsy symptom	1.162	0.28–4.85	0.837
Initial TN palsy symptom	0.743	0.14–3.94	0.728
Initial AN palsy symptom	0.487	0.04–5.62	0.564
Initial ON palsy symptom	0.286	0.01–10.02	0.49
1-year recovery rate of TN palsy	2.59	0.43–15.56	0.295
1-year recovery rate of AN palsy	0.44	0.02–10.05	0.61
1-year recovery rate of ON palsy	5.39	0.26–11.96	0.277
Distant metastasis-free survival			
Sex	4.36	0.44–26.62	0.316
Age	1.127	0.26–4.94	0.874
N stage	6.654	1.08–41.06	0.041*
Histological type	1.719	0.29–10.31	0.55
Treatment	0.536	0.13–2.25	0.395
Radiation dose	0.438	0.1–1.91	0.273
Tumor volume	0.809	0.17–3.71	0.785
Number of CN palsy symptom	1.39	0.24–7.63	0.735
Initial TN palsy symptom	1.91	0.34–10.78	0.464
Initial AN palsy symptom	1.552	0.13–18.68	0.729
Initial ON palsy symptom	0.798	0.22–20.34	0.972
1-year recovery rate of TN palsy	0.54	0.06–4.47	0.567
1-year recovery rate of AN palsy	2.29	0.12–43.43	0.58
1-year recovery rate of ON palsy	2.68	0.23–12.74	0.91
Locoregional recurrence-free survival			
Sex	0.807	0.07–9.74	0.866
Age	1.792	0.22–14.6	0.586
N stage	2.194	0.23–20.74	0.493
Histological type	2.21	0.2–24.47	0.518
Treatment	1.211	0.16–9.07	0.852
Radiation dose	2.794	0.38–20.67	0.314
Tumor volume	1.96	0.21–18.63	0.558
Number of CN palsy symptom	4.143	0.53–32.21	0.174
Initial TN palsy symptom	0.721	0.03–16.58	0.838
Initial AN palsy symptom	1.476	0.12–3.61	0.811
Initial ON palsy symptom	1.141	0.02–83.88	0.952
1-year recovery rate of TN palsy	7.559	0.37–153.2	0.188
1-year recovery rate of AN palsy	–	–	–
1-year recovery rate of ON palsy	–	–	–

CN, cranial nerve; HR, hazard ratio; 95% CI, 95% confidence interval; TN, trigeminal nerve; AN, abducens nerve; ON, oculomotor nerve

*P < 0.05

## Discussion

CN palsy due to tumor infiltration of cranial nerve implies the locally extensive lesion in NPC. According to the 8th edition AJCC staging system, CN palsy is classified as T4 disease. In the era of conventional radiotherapy and 3D-CRT, several studies reported number of CN palsy, palsy duration before treatment, recovery duration of palsy and complete recovery of CN palsy were associated with prognosis of patients with locoregionally advanced NPC [4, 5]. But there were lack of detailed information about the recovery process of CN palsy. This is the first study focus on the recovery process of CN palsy in patients received IMRT based definitive treatment.

Consistent with previous study, we found the most common CN palsy were trigeminal nerve palsy (90.4%), abducens nerve palsy (36.5%), and oculomotor nerve palsy (22.6%) in local advanced NPC [14]. We found that the longer duration of symptom was associated with the more severe symptoms, indicating more damage to the nerve. In this study, up to 77% of patients with TN, AN, or OS palsy could completely recovered within 2 years after treatment, indicating the efficacy of IMRT in complete recovery of upper CN palsy in NPC. AN and ON palsy in NPC were often due to intracranial involvement of tumor, such as involvement of cavernous sinus. Compared to IMRT, it was difficult to precisely deliver radiation dose to this area using conventional or 3D-CRT technique. IMRT has been shown to be associated with better 5-years local control compared with conventional RT and 3D-CRT [7, 15–17]. Therefore, the recovery of TN, AN and ON palsy might result from tumor ablation by IMRT. The recovery of HN and FN palsy were not investigated in this study due to low incidence of these symptoms. So it was still unclear whether IMRT could lead to better recovery rate for the two types of palsy compared with conventional or 3D-CRT technique.

We showed that the pretreatment grade of CN palsy was significantly associated with recovery process of CN palsy. All grade 1 palsy achieved complete recovery within 2 years after treatment. However, only 70% of patients with grade 2 palsy had complete recovery, although all of them had some improvement. The radical radiation dose of NPC was routinely up to 70 Gy in the tumor target for patients received IMRT [18, 19]. Even complete response was achieved after induction chemotherapy, the dose of radiotherapy was still up to more than 66 Gy in the target of primary tumor and lymph nodes [20]. In this study, the radiation dose, using median dose as dichotomy, was not associated with the recovery of TN and ON palsy. But for AN palsy, the recovery rate was much higher in patients received higher than 72.62 Gy. Recently, IC followed by CCRT was confirmed to be a more effective regimen than CCRT alone for locoregionally advanced NPC. The 3-year OS was improved by 5% with IC + CCRT [8, 9]. Therefore, we compared the recovery of CN palsy in patients treated with IC + CCRT to those treated with CCRT. We found that except for recovery of AN palsy in which IC + CCTRT had a trend to improve recovery, there were no significant correlation between recovery of CN palsy and treatment regimens. These results implied AN palsy might be relieved more easily in NPC patients after IMRT based comprehensive modality.

Previous study reported multiple CN palsy and long duration of pretreatment symptom were worse prognostic factors for OS in locoregionally advanced NPC in the era of conventional radiotherapy [5]. In this study, we found patients with more than 2 symptoms of CN palsy had worse prognosis for DMFS but not for OS. Several previous studies suggested that intracranial involvement of NPC might be potential routes for hematogenous dissemination due to anatomically rich venous plexus [21, 22]. In this study, 75% of patients with more than 2 symptoms of CN palsy had intracranial involvement (data was not shown), which may contribute to a higher risk of distant metastasis. Except the pretreatment duration of TN palsy was a prognostic factor for DFS and had potential predictive role to impact on the OS, the prognostic value of pretreatment duration of AN and ON were still unclear due to small sample size. Different from previous studies, we did not find prognostic value of complete recovery of CN palsy in this study. Advances in radiation technique and comprehensive treatment modalities might weaken the prognostic role of complete recovery for survival.

In multivariate analysis, consistent with previous studies, N stage was found to be a significant prognostic factor for DFS, DMFS and OS. We failed to detect prognostic value of CN palsy parameters for patient survival in multivariate analysis. Moreover, we could not find association of tumor volume with the number of CN palsy in this study (Additional file 6: Fig. S6), indicating tumor volume is not necessarily related to the extent of CN involvement in locoregionally advanced NPC.

## Conclusions

IMRT based comprehensive treatment could effectively promote the recovery of tumor-related CN palsy in locoregionally advanced NPC, especially for the TN palsy, AN palsy, and ON palsy. Pretreatment grade of CN palsy was significantly associated with recovery process of CN palsy. More than 2 symptoms of CN palsy was a poor prognostic factor for DFS in NPC patient. The prognostic role of complete recovery of CN palsy was not identified in our study.

## Supplementary Information

**Additional file 1: Fig. S1.** Dynamic changing of proportion of cranial nerve palsy after completion of treatment.

**Additional file 2: Fig. S2.** Survival analysis of patients with pretreatment duration within 2 months and more than 2 months of trigeminal nerve palsy. DFS, disease-free survival (a); OS, overall survival (b); DMFS, distant metastasis-free survival (c); LRFS, locoregional recurrence-free survival (d).

**Additional file 3: Fig. S3.** Survival analysis of patients with pretreatment duration within 2 months and more than 2 months of abducens nerve palsy. DFS, disease-free survival (a); OS, overall survival (b); DMFS, distant metastasis-free survival (c); LRFS, locoregional recurrence-free survival (d).

**Additional file 4: Fig. S4.** Survival analysis of patients with pretreatment duration within 2 months and more than 2 months of oculomotor nerve palsy. DFS, disease-free survival (a); OS, overall survival (b); DMFS, distant metastasis-free survival (c); LRFS, locoregional recurrence-free survival (d).

**Additional file 5: Fig. S5.** Survival analysis of patients with complete recovery and non-complete recovery from cranial nerve palsy. DFS, disease-free (a); OS, overall survival (b); DMFS, distant metastasis-free survival (c); LRFS, locoregional survival recurrence-free survival (d).

**Additional file 6: Fig. S6.** Correlation between tumor volume and number of cranial nerve palsy.

## Data Availability

All original data will be made available upon request.

## Abbreviations

NPC: Nasopharyngeal carcinoma
CN: Cranial nerve
AJCC/UICC: American Joint Committee on Cancer/Union for International Cancer Control
OS: Overall survival
3D-CRT: 3-Dimensional conformal radiation therapy
IMRT: Intensity modulated radiation therapy
IC: Induction chemotherapy
CCRT: Concomitant chemoradiotherapy
NCCN: National Comprehensive Cancer Network
CSCO: Chinese Society of Clinical Oncology
MRI: Magnetic resonance imaging
CT: Computerized tomography imaging
CTCAE: Common Terminology Criteria for Adverse Events
VMAT: Volumetric modulated arc therapy
PFS: Progression-free survival
DMFS: Distant metastasis-free survival
LRFS: Locoregional recurrence-free survival
SEM: Standard error of the mean
HR: Hazard ratio
TN: Trigeminal nerve
AN: Abducens nerve
ON: Oculomotor nerve
HN: Hypoglossal nerve
FN: Facial nerve
