# Supplementary figures and images for: Detailed analysis of recovery process of cranial nerve palsy after IMRT-based comprehensive treatment in nasopharyngeal carcinoma
Source: Radiat Oncol. 2021 Jun 27;16:118. doi: 10.1186/s13014-021-01846-x (PMC8237430; doi:10.1186/s13014-021-01846-x)

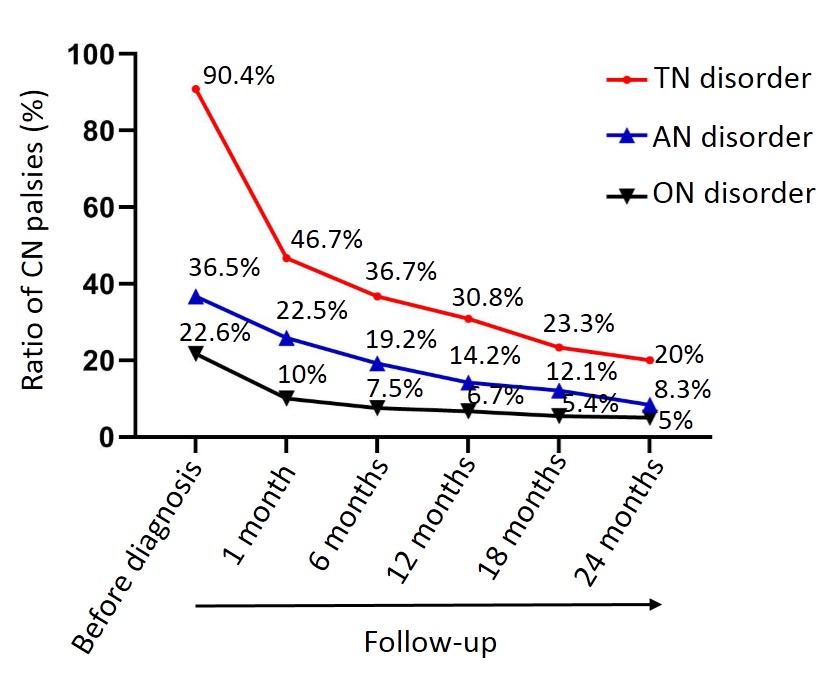

Supplement: Supplementary file 1 — Additional file 1: Fig. S1. Dynamic changing of proportion of cranial nerve palsy after completion of treatment. [file 13014_2021_1846_MOESM1_ESM.jpg]

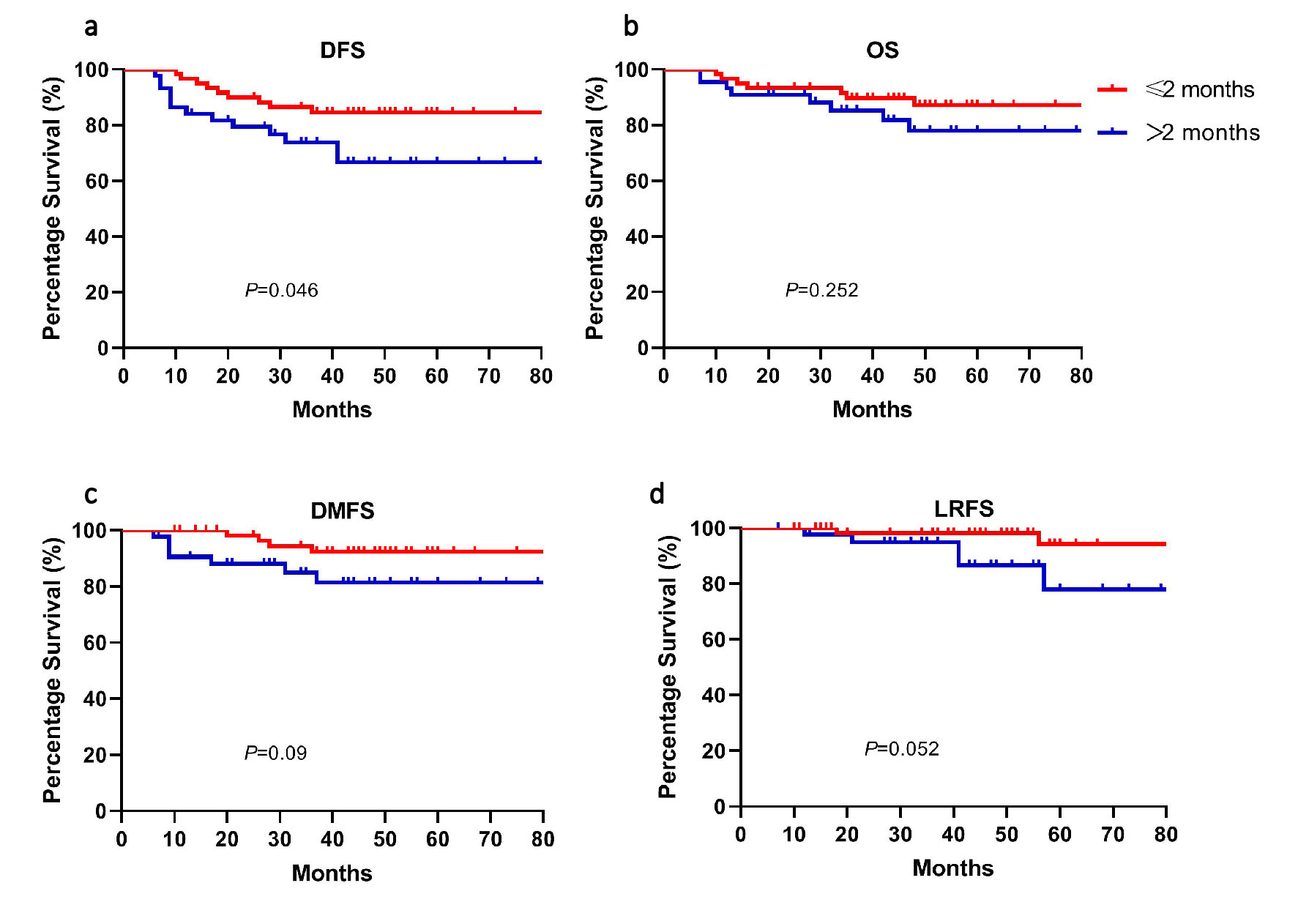

Supplement: Supplementary file 2 — Additional file 2: Fig. S2. Survival analysis of patients with pretreatment duration within 2 months and more than 2 months of trigeminal nerve palsy. DFS, disease-free survival (a); OS, overall survival (b); DMFS, distant metastasis-free survival (c); LRFS, locoregional recurrence-free survival (d). [file 13014_2021_1846_MOESM2_ESM.jpg]

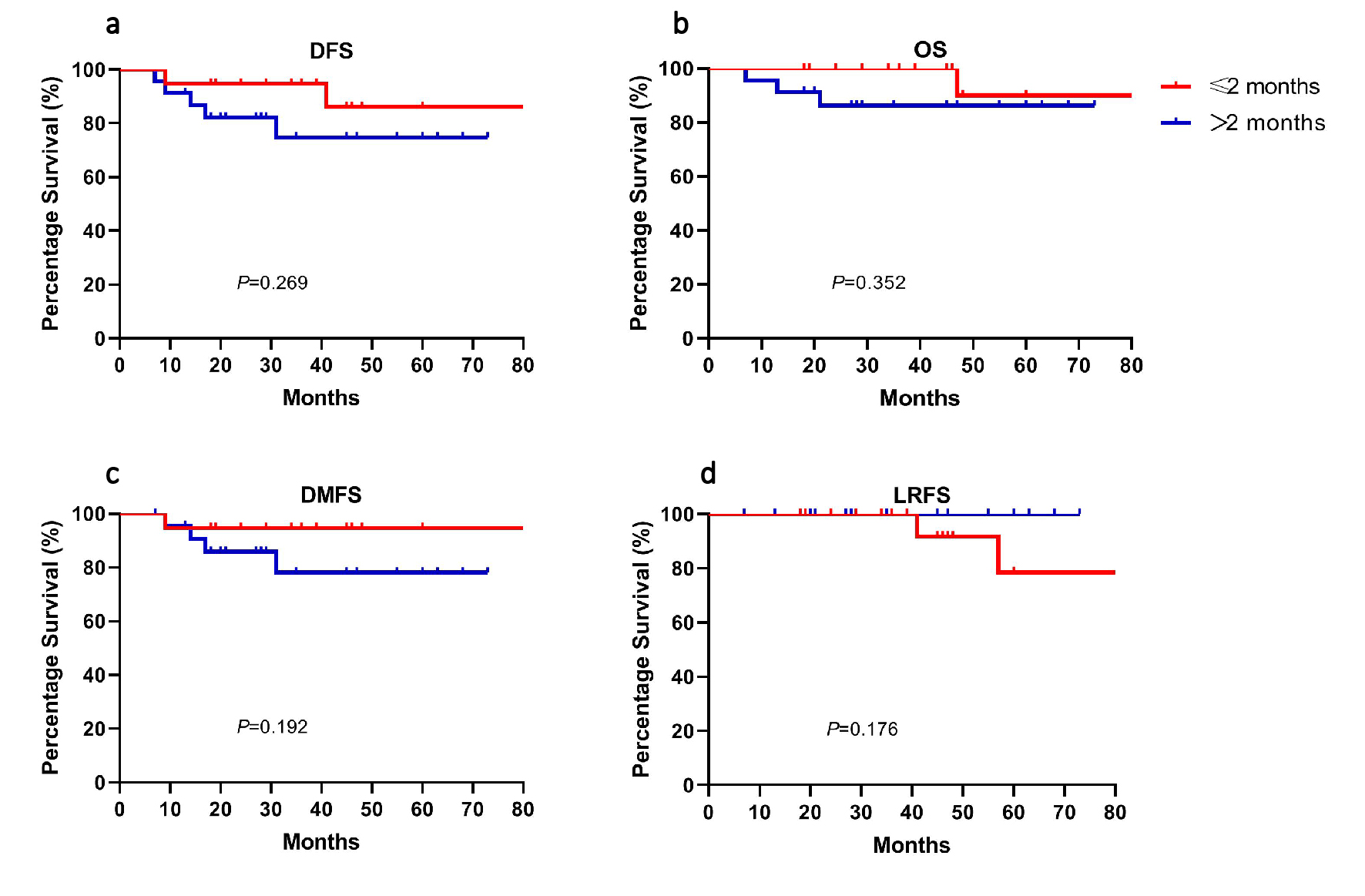

Supplement: Supplementary file 3 — Additional file 3: Fig. S3. Survival analysis of patients with pretreatment duration within 2 months and more than 2 months of abducens nerve palsy. DFS, disease-free survival (a); OS, overall survival (b); DMFS, distant metastasis-free survival (c); LRFS, locoregional recurrence-free survival (d). [file 13014_2021_1846_MOESM3_ESM.jpg]

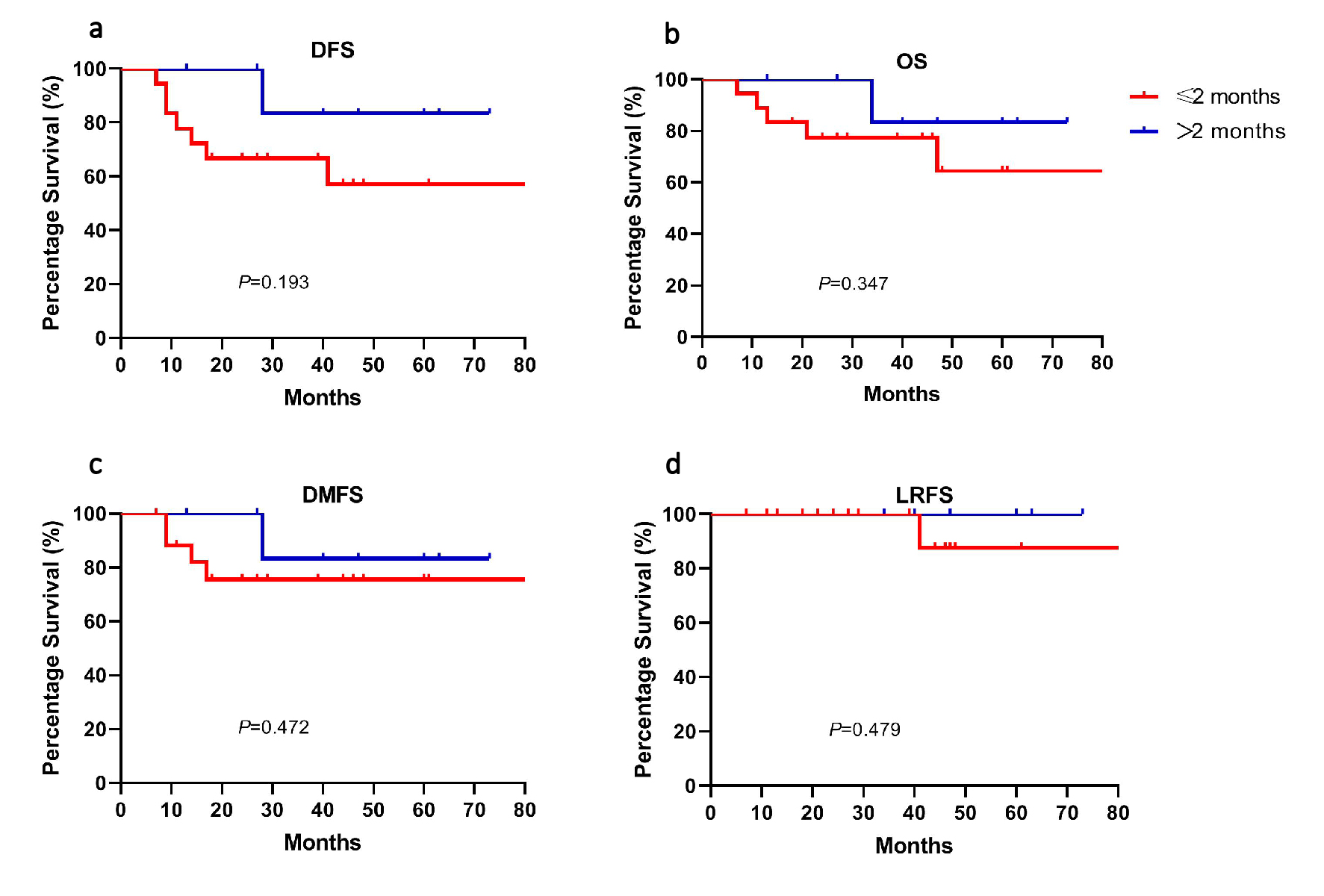

Supplement: Supplementary file 4 — Additional file 4: Fig. S4. Survival analysis of patients with pretreatment duration within 2 months and more than 2 months of oculomotor nerve palsy. DFS, disease-free survival (a); OS, overall survival (b); DMFS, distant metastasis-free survival (c); LRFS, locoregional recurrence-free survival (d). [file 13014_2021_1846_MOESM4_ESM.jpg]

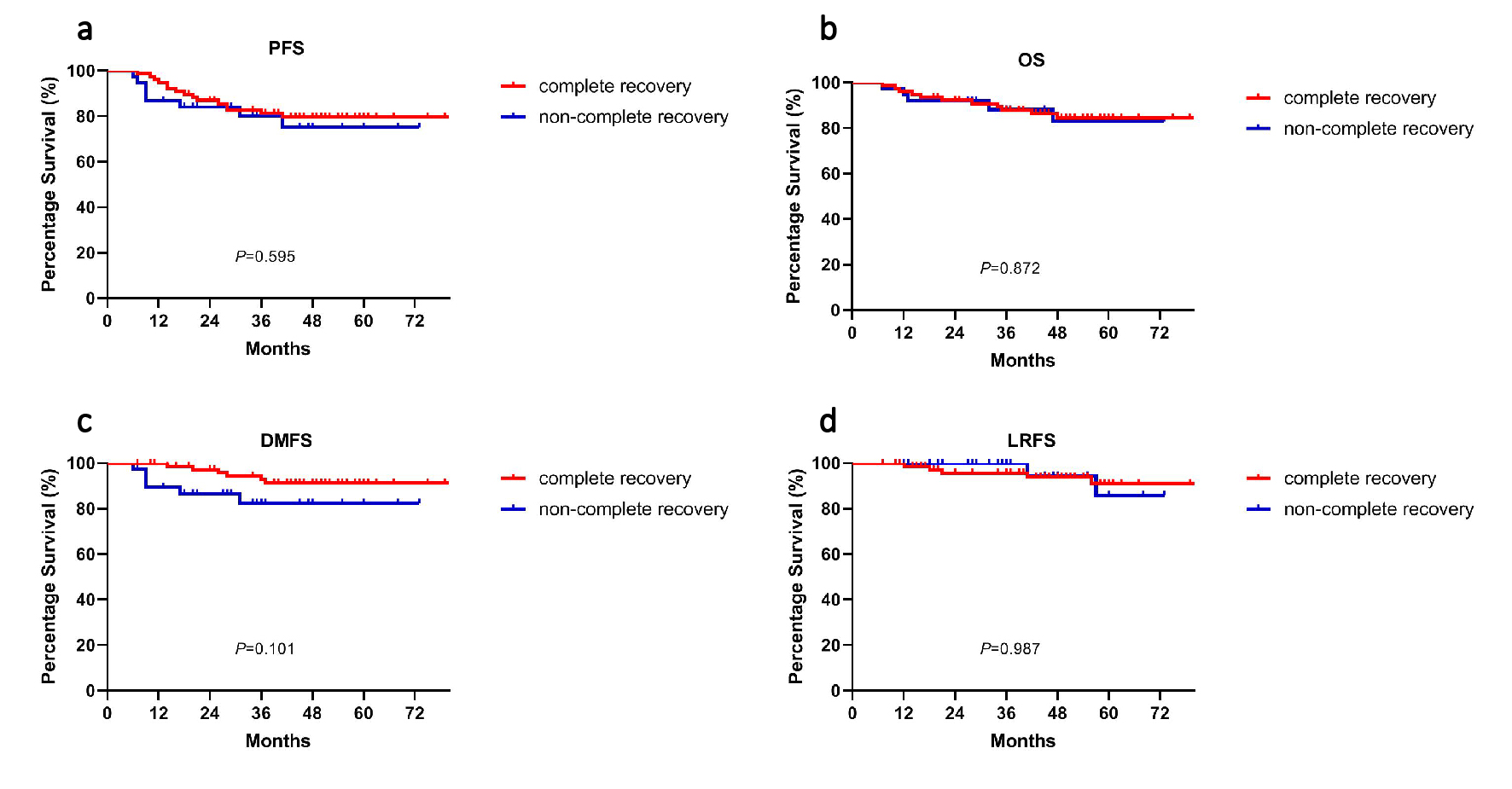

Supplement: Supplementary file 5 — Additional file 5: Fig. S5. Survival analysis of patients with complete recovery and non-complete recovery from cranial nerve palsy. DFS, disease-free (a); OS, overall survival (b); DMFS, distant metastasis-free survival (c); LRFS, locoregional survival recurrence-free survival (d). [file 13014_2021_1846_MOESM5_ESM.jpg]

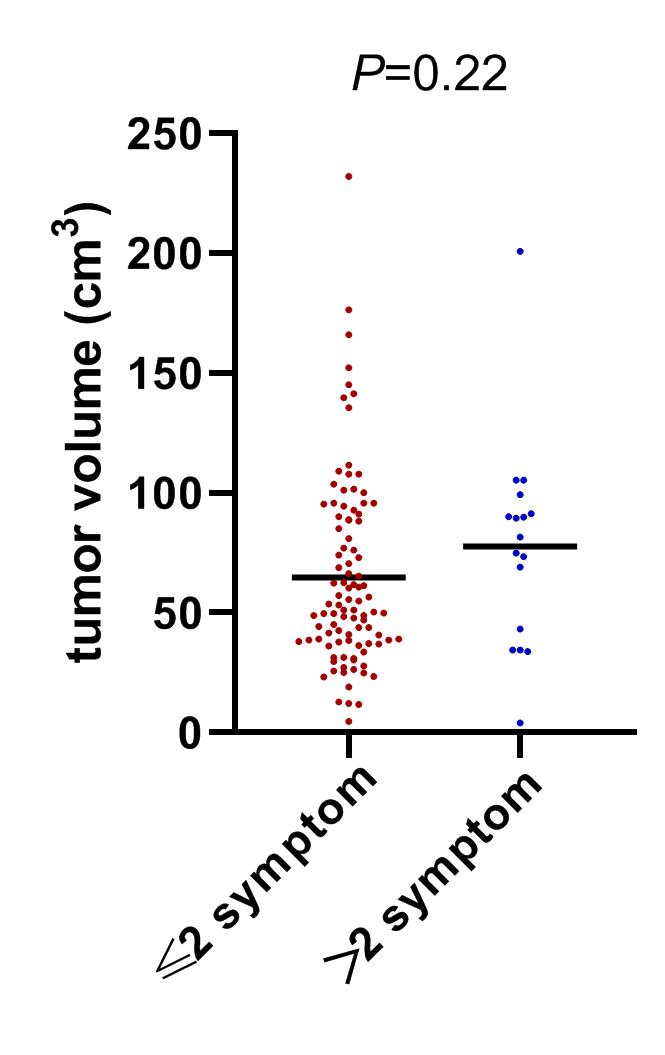

Supplement: Supplementary file 6 — Additional file 6: Fig. S6. Correlation between tumor volume and number of cranial nerve palsy. [file 13014_2021_1846_MOESM6_ESM.jpg]
